# Professional courage to create a pathway within midwives’ fields of work: a grounded theory study

**DOI:** 10.1186/s12913-021-06311-9

**Published:** 2021-04-07

**Authors:** Malin Hansson, Ingela Lundgren, Gunnel Hensing, Anna Dencker, Monica Eriksson, Ing-Marie Carlsson

**Affiliations:** 1 Institute of Health and Care Sciences, The Sahlgrenska Academy, University of Gothenburg, P O Box 457, 405 30 Gothenburg, SE Sweden; 2 Department of Public Health and Community Medicine, Institute of Medicine, Sahlgrenska Academy, Gothenburg University, Gothenburg, Sweden; 3 Department of Health Sciences, University West, Trollhättan, Sweden; 4 Department of Health and Nursing, School of Health and Welfare, Halmstad University, Halmstad, Sweden

**Keywords:** Work situation, Salutogenesis, Midwifery, Health promotion

## Abstract

**Background:**

The theory of salutogenesis focuses on resources for health and health-promoting processes. In the context of midwives’ work, this is not well described despite the importance for occupational health and the intention to remain in the profession. In order to promote a healthy workplace, it is necessary to consider the facilitating conditions that contribute to a sustainable working life. Therefore, the aim of this study was to explore health-promoting facilitative conditions in the work situation on labour wards according to midwives.

**Methods:**

A constant comparative analysis was applied to face-to-face interviews with midwives that constituted the empirical material in this classical grounded theory study.

**Results:**

The substantive theory of *Professional courage to create a pathway within midwives*’ *fields of work* emerged as an explanation of the health-promoting facilitative conditions in midwives’ work situation. The theory consists of a four-stage process with prerequisite contextual conditions: visualising midwifery, organisational resources and a reflective and learning environment, that were needed to fulfil the midwives’ main concern a *Feasibility of working as a midwife*. This meant being able to work according to best-known midwifery theory and practice in each situation. Positive consequences of a fulfilled main concern were a professional identity and grounded knowledge that enabled the development of the resistant resource professional courage. The courage made it feasible for midwives to move freely on their pathway within the different fields of work extending between normal and medicalised birth and being autonomous and regulated.

**Conclusions:**

A professional courage was required to create a pathway within midwives’ fields of work, to move freely depending on what actions were needed in a particular work situation. Professional courage could be seen as a resistance resource, enabling midwives to become resilient when dealing with the unpredictable work situation. However, there are vital organisational preconditions that needed to be fulfilled for workplaces to become facilitative, organisational resources, visualising midwifery and a reflective environment.

The theory can be used to foster health-promoting and sustainable work situations for midwives, which is possible if the organisational preconditions are met. This could be a key factor in retaining midwives in the profession.

## Background

In many countries, midwives experience strained work situations with high levels of work-related stress [1–3] and burnout [2–7]. There are various reasons cited for the prevailing heavy workload in the profession, such as insufficient work resources and understaffing [1, 4], as well as a poor organisational climate [1]. Furthermore, it has been suggested that clients place higher demands on midwives compared to other employees in the human service sector [6]. In Sweden, which is the context of this study, there are few studies about midwives’ work situations. Hence, there is a knowledge gap in line with the scope of this study. In 2013, it was found that one in three Swedish midwives had considered leaving the profession due to high levels of stress, a lack of staff and resources, conflicts at work, a low salary and, as a consequence, concern for their own health [8]. Dissonant views of midwifery and birthing – both from the midwives themselves and from other professions – can create contradictions in midwives’ professional roles and can contribute to midwives’ work situations in hospital settings being challenging [9]. Another Swedish study showed that midwives’ and nurses’ quality of life was affected by dissatisfaction with their work situation, while they also reported high levels of work-related exhaustion [10]. Risk factors for work-related stress and burnout have proven to be a young age and little work experience [2, 7, 8].

However, a work situation with sufficient resources can contribute to positive health development [11]. The theory of salutogenesis [12] focuses on resources for health and health-promoting processes with the core values of equity, participation and empowerment [13]. In the Ottawa Charter for Health Promotion [14], health was described as a resource in everyday life, not as an absence of disease, and health promotion was viewed as a process of enabling people to gain control over health determinants and achieve equity in health. The term health-promotion is in this study related to the midwives’ work situation and defined according to the WHO definition as a process of enabling people to increase control over, and to improve their health [14]. The term facilitative is regarded as enabling conditions in the work situation, which promote a healthy working life for midwives. Both health-promoting research and activities have been criticised for the lack of a sound theoretical foundation [13] and salutogenic theory is one theory that has been suggested to fill this gap [13]. Thus, salutogenic theory and health-promotion is the theoretical foundation of the present study.

A sense of coherence (SOC), the key concept in salutogenic theory, is a global orientation, and is not merely a scale for measurements. It is based on cognitive, behavioural and motivational dimensions reflecting the interaction between the individual and the environment; in this study, the midwives and their work situations. An individual with a strong sense of coherence perceives life as comprehensive, manageable and meaningful, and is confident about his or her own external and internal resources [12, 15]. The generalised and specific resistance resources provide the prerequisites for developing a strong SOC [16]. If one has an absence of resources, this is perceived as a deficit and can lead to stress if this is not balanced by other resources [17].

The workplace is one of the most important settings for health promotion due to its effect on physical and mental health [18]. The workplace also influences social and economic well-being and, in turn, the whole of society [19]. The salutogenic health-promoting ideas assume that each individual, workplace and organisation has resources that can be used to preserve and develop health and a strong SOC [12] and therefore has the potential to retain midwives in their profession. One integrative review [20] stated that midwives valued their colleagues and felt a passion for midwifery and for protecting normality which belongs to the midwives professional domain. The results also showed that there was research focusing on why midwives leave the profession, but there was a lack of studies focusing on health-promoting conditions or having a salutogenic approach.

Therefore, the aim of this study was to explore health-promoting facilitative conditions in the work situation on labour wards according to midwives.

## Methods

### Study design

The aim stated above is consistent with the open approach of classical grounded theory (CGT) methodology used in this study [21]. CGT allows the data to guide and create conceptual theories of social processes. Salutogenic theory [12] is used as an approach to focus on the health-promoting factors in midwives’ work situation and is also interlaced with the emerged theory in the discussion section.

### Participants

The target group was midwives working on five different labour wards in one university hospital and two regional hospitals in the Västra Götaland Region in Sweden. Approval to conduct the study in the different workplaces was obtained from the heads of department for obstetrics and the heads of the units in the different hospitals. Purposive sampling was used and written information about the study was sent to the different labour wards. The inclusion criteria were being able to understand and speak Swedish and having at least 1 year of work experience as a midwife on a labour ward. Midwives interested in participating announced their wish to participate to the first author (MH) through e-mail or via the telephone. When in contact, both written and oral information about the study design and aim was given. The participants were also informed that their participation was voluntary and that they could withdraw their participation at any time without giving any reason. Before the start of the interview, the participants gave their informed written consent and were assured that the data would be treated according to EU General Data Protection Regulation (GDPR).

Twelve midwives were interviewed. They were all women, ten were co-habiting with a partner, and seven had at least one child living at home. The midwives had work experience in their current workplace of between 2 and 34 years (Table 1).

**Table 1:** Characteristics of the participants

		Age	Years as a midwife	Work experience in current workplace
N	Valid	12	12	12
Mean		56	22	17
Median		58	24	21
Range		36–64	2–38	2–34

### Data collection and analysis

The data was collected between October 2019 and February 2020. Individual face-to-face interviews were conducted by the first author (MH). The interviews were undertaken according to the participants’ wishes, predominantly in the workplace, but also in the participants’ homes or at an undisturbed place chosen by the participants. The interviews were audio taped and lasted between 52 and 88 min, with a mean length of 69 min.

The data collection focused on salutogenic health-promoting facilitative conditions in the midwives’ work situation. However, sequential questions were asked based on what emerged during the interviews. The interviews started with a salutogenic open-ended question, “What is good in your work situation?” and this was followed by different open-ended questions about factors of importance to promote a sustainable working life. Reflective memos were produced after each interview by the first author (MH). Due to the CGT methodology, the data collection and analysis took place simultaneously [22]. This means that previous interviews and codes that emerged from the analysis led to supplementary and comparative questions in the following interviews through an abductive process.

The analysis started at an empirical and descriptive level and continued with a conceptualisation via code, compare, categorise and conceptualise to reach a more abstract and conceptual perspective and theory [23]. The analysis began during the transcription of the interviews, and memos were written continuously throughout the analytical process [24] by the first author (MH).

Open coding was done line by line and separately by the first (MH) and last (IMC) author. Open coding was guided by asking the data “what was happening”. Codes were named as gerunds (keeping codes short, precise and analytical) and was kept close to the data. The next step was comparing the codes with similar content and categorising them together into concepts with a higher abstraction level, thus revealing the emergent social-pattern conceptualisation. Through the constant comparison of the concepts that appeared, the main concern emerged on the basis of abstraction [25–27]. The coding was an abductive process, moving back and forth between parts and the whole, and between indicators and concepts. Consequently, both inductive and deductive approaches were used, which is customary in CGT [23, 27]. Memos, which were descriptive in the beginning, evolved into ideas of relationships between concepts, and the first author (MH) analytically interpreted the data and the emergent social patterns during the analysis process [24]. When the main concern and an initial theory emerged, selective coding was applied – only coding data that was relevant to the main concern – to develop the theory further and to saturate the concepts [24, 25]. The second author (IL) participated in this part of the analysis. By writing memos upon memos and sorting them carefully, the theoretical codes emerged, which further developed the theory [23, 27, 28]. Theoretical sampling was used, and data collection continued until theoretical saturation was reached; that is, when no data was found that developed the concepts or theory further [22, 25]. The abductive process, during data collection and analysis, permits the researcher to achieve a grounded theory with theoretical sensitivity through being close to the data, and applying constant comparisons and conceptualisations [25]. The remaining authors (GH, AD and ME) participated in the later phases of the analysis in relation to the comprehensibility of the substantive theory as well as in their areas of expertise (specified in the Contribution of authors).

CGT aims to generate conceptual theories, abstracted from the time, place and people. The social process and patterns of human behaviour are categorised, rather than individual people; therefore, personal quotes are not used [23, 25, 29].

## Results

During the analysis conditions emerged that facilitate a health-promoting working life for midwives and they resulted in the substantive theory of *Professional courage to create a pathway within midwives’ fields of work.* A four-stage process of health-promoting facilitative conditions in midwives’ work situations built up the emergent theory. The first stage conceptualises the prerequisite conditions: *Visualising midwifery, Organisational resources* and a *Reflective and learning environment* that were identified as necessary to fulfil the second stage’s main concern: *Feasibility of working as a midwife*. The third stage presents the health-facilitating consequences of a fulfilled main concern with acquired grounded professional knowledge and professional identity that led to an attained *Professional courage,* which can be seen as a resistance resource to facilitate sustainable occupational health*.* This courage, in turn, led to the possibility of being able to create a pathway within midwives’ fields of work. The substantive theory of attained *Professional courage to create a pathway within midwives’ fields of work* will conclude the results section after going into more detail about each part of the theory.

### The main concern: feasibility of working as a midwife

*Feasibility of working as a midwife* constitutes the main concern for midwives, which, when fulfilled, facilitates a health-promoting salutogenic, manageable and meaningful work situation. Feasibility means being able to work in an evidence-based manner; that is, to rely on current research and best practice in each situation. To be trusted and listened to by other professionals, managers, colleagues and the birthing woman and her partner was perceived as a necessity for midwives’ work to be health-promoting. This meant that there was confidence in the midwives’ knowledge and actions and an acknowledgement of their professional standpoints. Being trusted also meant that they were able to work autonomously as midwives based within their professional domain. Having influence over one’s own work made it feasible to work as a midwife by not having to involve obstetricians if that was not called for, and by not being controlled and regulated by the medicalised care in the professional domain of normal births.

Midwives strived to be of professional importance and to be of use both to the birthing woman and her partner and to other midwives, as well as to the team around the birthing woman. Midwives pursued high-quality care and strove to keep the birthing woman and her desires in focus, trying to care for one woman at a time. When it was possible to work in the stated manner, it was perceived as a health-promoting facilitative condition in the work situation that made the work manageable. The midwives depicted the midwifery profession character traits as strong, dedicated, enthusiastic and committed to the work, and also as striving to make a difference and to satisfy everyone’s needs – both the birthing women with their partners and their colleagues. These characteristics were positive when achievable and negative for the midwives’ work situations when they were unattainable. The midwifery profession was also depicted as isolated, as shielded from the other professions and social interactions. Hence, inhibiting social processes such as cooperation and communication between the different individuals and professions was seen as a deficit. There were no clear guidelines or descriptions for the midwives’ professional domain of normal births, which led to midwives’ work becoming unclear and invisible for themselves and for others. The medical aspects were, on the other hand, thoroughly described and regulated with memorandums and regulations, and were therefore more predictable and clearer.

#### Prerequisite conditions to fulfil the midwives’ main concern

**Fig. 1:**
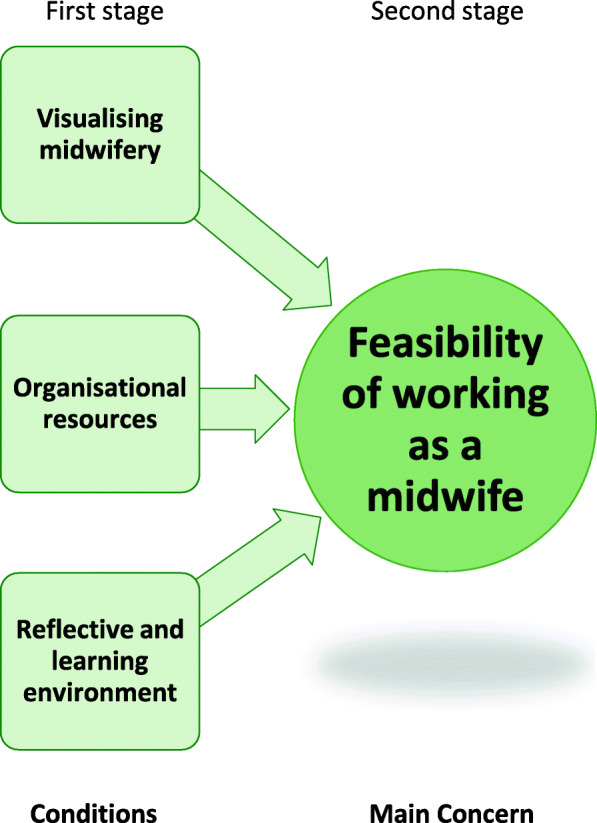
The prerequisite conditions to fulfil the main concern: *Feasibility of working as a midwife*

In order to be able to achieve the feasibility of working as a midwife and to have the prerequisites for a healthy and sustainable work situation, certain conditions needed to be fulfilled to the greatest extent possible. This was needed in the organisation and in the social interaction between the different individuals and professions to enable a healthy workplace. The conditions were: *Visualising midwifery, Organisational resources* and a *Reflective and learning environment* (Fig. 1). Fulfilled, they were seen as health-promoting facilitative conditions and they led to the feasibility of being able to work as a midwife.

The importance of *Visualising the midwifery* profession in practice and in the organisation, was emphasised by the midwives. The visualisation was eligible on three levels: individual, group and societal. At the individual level, the individual visualisation of the midwife working on the floor as an expert during normal births, and of what that work entails both in practice and theory, was of note. At the group level, the professional role of midwives needed to be clear and visual in the organisation of care and for the other professions. At the societal level, the midwives stated that the profession needed to be visual, acknowledged and recognised in the discourse in society regarding midwives’ professional responsibility for normal births. Visualising midwives’ work and scope of practice was seen as enabling professional autonomy, where midwives could take on their professional responsibility for normal births. A strong sense of autonomy facilitated resilience and midwives coped even in strained work situations. Visualising midwifery also made it feasible to establish a clear professional identity within the medicalised care that was expressed as prevailing in the studied context. When midwifery was visualised, there were more distinct structures and decision-making paths. Midwives strived to be advocates for normal births. When midwives’ professional domain of being responsible for normal births was clear in the organisation and in the inter-professional team, then it facilitated clarity in roles and was positive for teamwork, and conflicts could be avoided. Thus, a well-functioning and health-promoting social process prevailed. Midwives expressed how everything that was normal belonged to the midwife and it was of outmost importance that the midwifery profession and their work was made visible, especially since most of the practical work was isolated behind closed doors in birthing rooms. On the other hand, when midwifery was invisible in a practice dominated by a medical view, a loss of professional identity was present. Then midwives had a feeling of being obstetrical nurses rather than midwives with the responsibility for normal births, which belongs to their professional domain. Consequently, clear professional roles were required for visualising the midwifery profession and achieving an appropriate division of labour.

Another necessary condition was sufficient *Organisational resources* to make it feasible and manageable to work as a midwife. When there were adequate staffing and time resources, midwives could devote time to the individual woman and couple, being in the room continuously based on the couple’s wishes. Then the midwife had the possibility of performing his or her professional skills and could be proud of them. Consequently, midwives could let go of the ward ‘outside’ and be present in the birthing room. Stability in the organisation and management was also noted as key to enabling work in a health-promoting context. Being able to practise as a professional midwife with the responsibility for one woman, being with the woman and continuously supporting her in a present manner and not running between birthing rooms were important objectives for the midwives to enable them to perceive a health-promoting work situation. Organisational resources were described as a vital condition for the main concern and as a health-promoting facilitative condition in the work situation, when fulfilled. Then the organisation caters to the standards of high-quality care and the well-being of its employees. On an individual level, basic needs like having lunch, going to the toilet or having time to drink a glass of water were appreciated and were seen as personal health-promoting facilitative conditions, which in turn supported the midwives’ abilities to provide adequate care. With sufficient resources, midwives could let go of their fear of doing something wrong, of causing harm and of endangering patient safety.

A third condition identified as important for a health-promoting work situation was a *Reflective and learning environment*. Constructive communication and an adaptability, openness and equality between the inter-professional teams facilitated the desired work environment and a trusting solidarity, which in turn promoted a reflective and learning environment. Nevertheless, at the same time, the midwives needed time together within the profession to strengthen midwifery, not to exclude other professions, but to evolve into being experts in the normal birthing process. This professional reflection facilitated midwifery work and balanced the medicalised view of birth. This environment also led to an ability to pass on knowledge, experience and midwifery skills; moreover, it eased the tension of the movement between midwives’ fields of work (visualised in Fig. 3 and further conceptualised below). Senior midwives emphasised that they had an important role in building this environment, by passing midwifery skills and their attained grounded knowledge on to junior colleagues. This gave them the ability to stand up for the midwifery profession and to be able to let go, which meant letting go of guilt, self-criticism and leaving their working life at work, hence having a good work–leisure balance. An invigorating work climate with support from colleagues as well as managers was a significant key to achieving a reflective and learning environment and good psychosocial interaction. A reflective dialogue, both midwife to midwife and in the inter-professional team around the birthing woman, was required to enhance reflexivity, which in turn improved the possibility of meeting the demands of the workplace context.

### Consequences of a fulfilled main concern

**Fig. 2:**
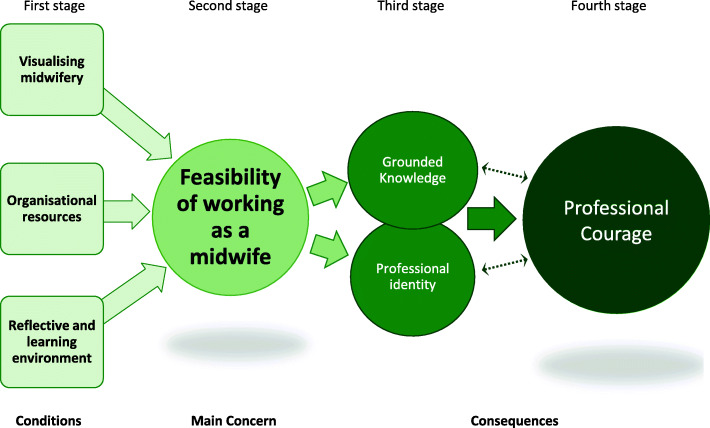
The four-stage process of health-promoting facilitative conditions in midwives’ work situation

When the prerequisite conditions of organisational resources, visualising midwifery throughout the organisation and a reflective and learning environment were met, the *Feasibility of working as a midwife* was conceivable*.* Then midwives had the ability to focus on their professional domain in a meaningful way*.* This, in turn, led to positive consequences in the third stage of the process (Fig. 2) of midwives having the possibility to form a professional identity that was based on a mutual professional grounded knowledge in both practice and theory. This common knowledge in the profession is visible and known regardless of the individual situation. Furthermore, the midwives then felt secure and confident in their acquired professional identity and embodied grounded knowledge and could attain *Professional courage*, further conceptualised below. This professional courage in the fourth stage also led to a positive spiral of deepening the grounded knowledge and professional identity that, in turn, intensified professional courage in a mutual interplay. However, the midwife had to attain grounded knowledge and a professional identity before professional courage could primarily be reached. Nevertheless, to be able to develop professional courage, the earlier stages in the four-stage process needed to be fulfilled.

### Professional courage within midwives’ fields of work

**Fig. 3:**
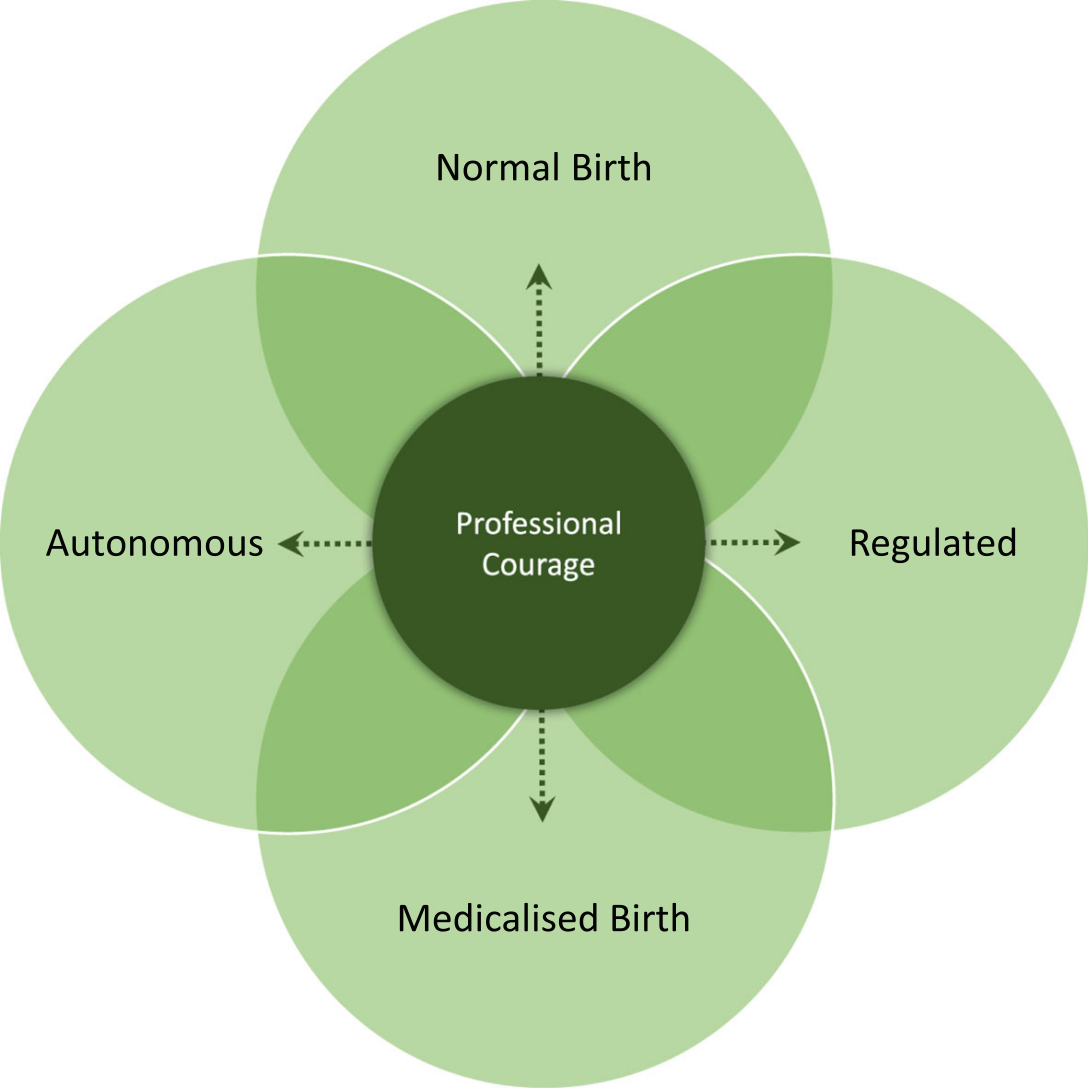
The health-promoting facilitative condition of attained *Professional courage to create a pathway within midwives’ fields of work*

When the positive consequences of an acquired grounded midwifery knowledge and professional identity were met, then the midwives could attain *Professional courage to create their pathway* and move *within midwives’ fields of work* (Fig. 3) in a floating manner and with enhanced reflexivity. This meant having intrinsic autonomy and one’s own responsibility as a professional midwife for normal births, instead of being a regulated medicalised obstetrical nurse.

Midwives’ fields of work here represent the professional domain and midwives’ areas of knowledge. It extends over a continuum from handling normal births, which is the independent professional area, to handling medicalised births, which requires involvement and collaboration with obstetricians. Thereby, midwives have to handle work situations that are both autonomous and regulated.

Midwives expressed how they strove to be autonomous in their area of expertise – normal births – and in the profession. At the same time, they are regulated in both normal and medicalised births by the prevailing medicalised care that focuses on the medical and risk factors of birthing. When such regulation occurred during normal births, midwifery skills and clinical experience became inferior in relation to the medical dominance, and midwifery work then became invisible. Childbirth can be both a natural physiological event as well as medical event, and this means that midwives must take control in uncontrolled work where the outcome is unpredictable. The participants depicted this as taking a leap into the unknown during every step and during every day at work.

To make it possible to use the pathways between the fields of midwives’ work, developing *Professional courage* was, according to this theory, required. Professional courage can be seen as a resistance resource, which makes the midwife resilient when dealing with unpredictable work situation. This courage allows the midwife to navigate and move freely in any direction along the pathways, depending on what actions are needed in a particular work situation. The midwives disclosed how actions and decisions illuminated how they should create their own pathway within the midwives’ fields of work due to the context and given situation. However, they need to master their fear of doing something wrong and gain professional courage to be able to traverse the pathways between the different fields of midwives’ work to avoid an erosion of the midwifery profession and instead strengthen midwifery and normality. It is conceivable to be an advocate for normality, regardless of the situation, when professional courage is attained in the work situation. When midwives stood up for the midwifery profession, they experienced their work situation as health-promoting. Their professional courage generated a resistance, which facilitated the midwives’ main concern of being able to work as a midwife. The courage supported the midwives in creating a pathway in a work situation that is both autonomous and regulated, normal and medicalised. Consequently, having professional courage facilitated a health-promoting work situation but the organisation needed to fulfil certain prerequisites and relevant contextual conditions for this to unfold.

### The theory of attained professional courage to create a pathway within midwives’ fields of work

**Fig. 4:**
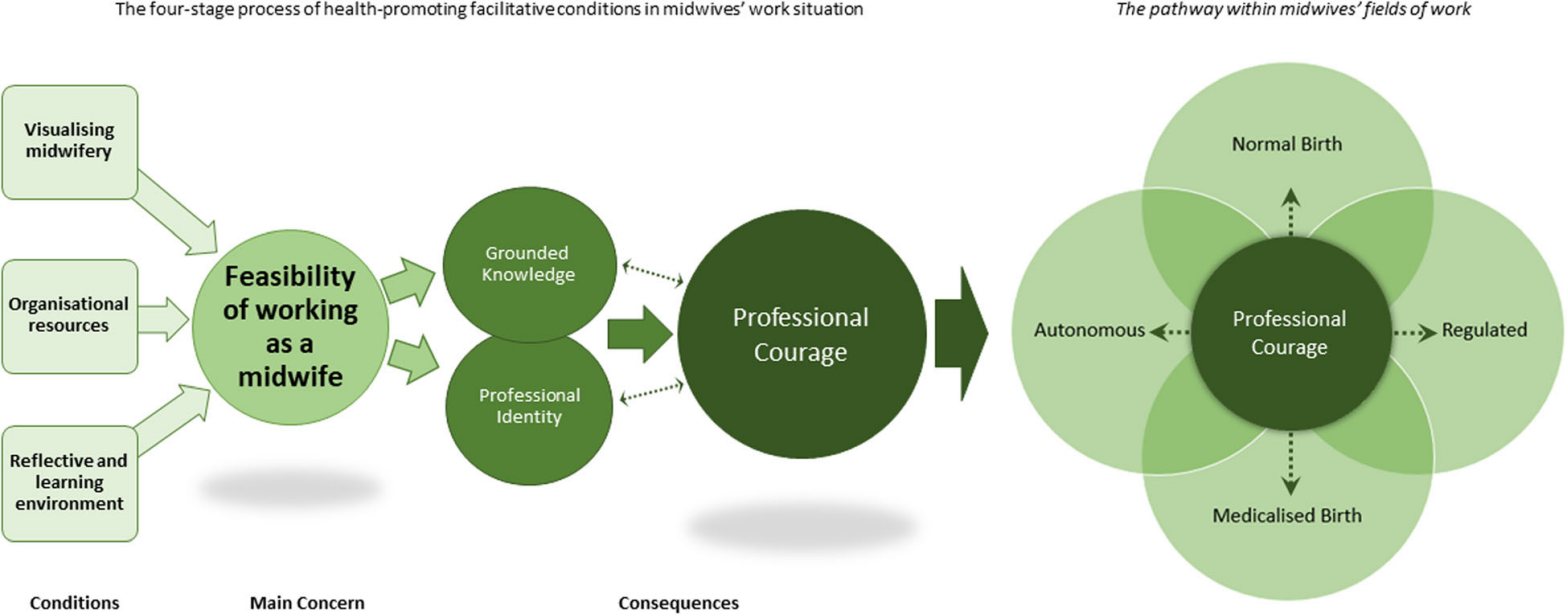
The theory of attained *Professional courage to create a pathway within midwives’ fields of work*

The substantive theory of attained *Professional courage to create a pathway within midwives’ fields of work* emerged as an explanation of health-promoting facilitative conditions in midwives’ work situation. The theory consists of a four-stage process (Fig. 2) where the first stage involves providing the required contextual organisational conditions: *Visualising midwifery, Organisational resources* and a *Reflective and learning environment*. When these conditions were met, it led to the second stage, where it was conceivable for the midwives to experience the *Feasibility of working as a midwife,* which was their main concern. The feasibility of working as a midwife means having the prerequisites in place to work according to the best-known midwifery theory and practice in each situation, which was perceived as a conclusive health-promoting facilitative condition. The third stage consists of the positive consequences of a fulfilled main concern – *Professional identity* and an attained *Grounded knowledge –* that enabled the fourth stage, the development of a resistance resource: *Professional courage.* This courage, in turn, made it feasible *to create a pathway within midwives’ fields of work* at any given moment and still maintain good occupational health and a sustainable work situation, despite the latter’s unpredictability. Consequently, this theory explains the process of health-promoting facilitative conditions in midwives work situation that leads to developing professional courage to enable midwives to create a pathway within their fields of work (Fig. 4). According to the theory, and based on an analysis of interviews with midwives on labour wards, the four-stage process must be realised to enable the resistance resource of professional courage.

## Discussion

### The substantive theory explains the process of health-promoting facilitative conditions in midwives’ work situation

The aim of this study was to explore health-promoting facilitative conditions in the work situation of labour ward midwives, hence having a salutogenic health-promoting approach. The substantive theory of attained *Professional courage to create a pathway within midwives’ fields of work* emerged as an explanation of a process of health-promoting facilitative conditions in midwives’ work situations. When fulfilled, the four-stage process of facilitative conditions led to professional courage that can be seen as a resistance resource in the salutogenic model of health [30]. This courage contributed to sustainable occupational health, where midwives felt empowered, competent and resourceful, as well as having the assets to handle their work situation. Then they had a possibility of developing their profession and standing up for normal births. Another Swedish study found similar results and suggested that the important factors for work satisfaction among midwives and nurses were the possibility of being able to make use of and develop professional competence [10].

The concept of professional courage was according to this study a prerequisite that made it conceivable to find a health-promoting way of handling the complex and constant changing work situations and managing the movement of creating a pathway between midwives’ fields of work. This professional courage can be interpreted as facilitating a balance between the salutogenic resources and deficits in the work situation [11] as well as a balance between the different fields of midwives’ work. Further, the findings show that courage led to resistance against being over-medicalised, and the midwives could create a pathway between normal and medicalised births as well as between being autonomous and regulated.

The present substantive theory relates to the three important SOC concepts: comprehensible, manageable and meaningful work situation [15, 31]. The result of visualising the midwifery profession on an individual, group and societal level can be seen as making the organisation of care more comprehensive for the inter-professional team around the birthing woman. Comprehensibility is described as an important work resource for the possibility of being able to perceive a SOC in the salutogenic theory [11, 16]. When the organisation clearly visualised the midwifery profession as being responsible for normal births, there were more distinct decision paths and clear roles in the inter-professional team around the birthing woman in this study. This could be understood as making the work comprehensive and enabling a SOC related to the work situation [11].

One other aspect of the theory was the ability to work autonomously, which has been found in earlier studies to enhance the capacity to cope with the environment even when it is strained [32]. To be autonomous in work situations can be related to meaningfulness and motivation in regards to work commitment [12]. Creating the pathway between midwives’ fields of work was a health-promoting resource that led to a feeling of expertise. It is known that autonomy is a job resource that leads to high levels of work engagement [11]. This engagement can, according to Gregor et al. [11], in turn lead to a motivational process that buffers the health-impairment process and job-demand deficits, and strengthens job resources. This is supported by a qualitative study that states that practising autonomously increases midwives’ resilience and their feeling of usefulness [33]. According to the salutogenic model, there is a need for motivational factors [16], such as autonomy presented in this substantive theory, to access the resistance resources. On the other hand, the findings from this study showed that there was a work overload and time pressure that may inhibit access to these resistance resources [16].

Sufficient organisational resources is, in this substantive theory, interpreted as leading to an experience of balance between demands and resources and a feeling of manageability. This is supported by Nilsson et al. [34], who found manageability to be a work-related, specific enhancing resource that increased the employees’ control over their work situation. The interviewed midwives expressed an often-experienced lack of human resources. They were understaffed, but when the employer took control of those factors and created conditions that balanced demands and resources, the midwives experienced a health-promoting workplace for the individual, for the group and within the organisation [35]. Such a salutogenic and sustainable work situation can in turn affect the performance of high-quality care. In the salutogenic theory, job satisfaction and commitment are described as positively related to resources in the organisation as well as to the intention to stay in a job [31].

A reflective and learning environment and the feasibility of working as a midwife were noted to create meaningfulness in the work situation and in the profession. During education, midwives pass on knowledge and midwifery skills to the students by a reflective dialog and this was continuously an important part of building the profession to enhance reflexivity and being able to meet the demands in the work situation.

According to the present substantive theory, professional courage and a strong sense of autonomy in the profession are health-promoting and could be understood as facilitating resistance against work stress [16]. When midwives had a strong professional identity and developed professional courage, they seemed to be able to resist the role of regulated medicalised obstetrical nurses, and instead felt confident in their own abilities and were advocates for normality and midwifery. This result corresponds with a study [36] about midwives’ professional identity that was described as a negotiation between the external medical model (obstetrical nurse) and the internal definition (professional midwife) connected to the midwives’ philosophy of normal births. It also complies with Hunter and Warrens’ [37] results of midwives’ workplace resilience when they experience professional belonging and a professional identity.

Women-centred care is interrelated with the salutogenic approach, according to studies from Europe, and is a main goal in midwifery as well as a theoretical perspective [38, 39]. In this study, women-centred care was seen as an organisational resource to achieve a health-promoting work situation. Although one-to-one care is not offered in Sweden due to the organisation of care, where midwives often care for more than one woman, midwives in this study emphasised that one-to-one care was a health-promoting way of working. On the other hand, medicalised care was considered a deficiency in the work situation. Medicalisation has, according to Johansson et al. [40], led to the increased involvement of obstetricians and to medical interventions becoming routine in normal childbirth.

The medicalised power structure has generated an overregulation of midwives’ work with a focus on risk factors, where the midwives’ scope of practice has become inferior and invisible, according to the midwives. This may be seen as a generalised resistance deficit [17]. This is in line with Larsson et al.’s findings that increased medicalisation and organisational changes lead to midwifery handcraft skills and clinical experience being less valued and that this could enhance feelings of a loss of control [41]. The midwives in the present study could when medicalised feel trapped in the regulated field, unable to create a pathway to the other fields of midwives’ work. This generates resistance from the midwifery profession against medicalised care and against the insufficient room for manoeuvring due to the power hierarchy between midwifery and the prevailing medicalised care. Working between different belief systems and philosophical views on birth can be problematic [42] and, according to the results in this study, midwives must be able to transact a pathway between the different fields of midwives’ work to enhance reflexivity and to be able to exercise their scope of practice.

To attain *Professional courage* and to be able to *create a pathway within midwives’ fields of work,* there were preconditions that needed to be fulfilled in a four-stage process to deal with the midwives’ main concern: *Feasibility of working as a midwife*. A workplace that provides a supportive social and physical environment and that enables the feasibility of working as a midwife could be seen as a workplace that enabled resources. In a strained and unpredictable work situation, the resources may prevent the tension from being transformed into stress [43]. Midwives in the present study expressed how they placed high demands on themselves and on the profession. However, if the profession was visualised and they had strong support and high levels of control in a reflective and learning environment that provided organisational resources, it was perceived as manageable. When a work situation is comprehensive and manageable, then positive developments in the work situation and health are possible, according to Gregor et al. [11].

It is often assumed and demanded from the organisation that a midwife should develop a *professional identity* and *grounded knowledge* without the prerequisite conditions in the work situation. Thus, demands are made solely of the individual rather than the organisation. The organisation needs to cater for high-quality care and the well-being of its employees; it should not be up to the individual midwife. Hence, the organisation needs to provide the right working conditions and cherish as well as visualise the midwives’ competence. This is in line with a mixed method study that states that midwives’ work in a strained context with lack of support for the supporter and that it could be beneficial to implement midwifery models of care to strengthening midwifery practice [44].

Taking the theory of *Professional courage to create a pathway within midwives’ fields of work* to a practical level, we suggest that the emerged organisational resources need to be adapted to the job demands on labour wards, including the midwives’ feasibility of doing their work in accordance with theory, evidence and best practice. Even more concretely, this would mean that the organisation utilises and strengthens the health-promoting facilitative conditions: *Visualising midwifery*, *Organisational resources* and a *Reflective and learning environment*. That, in turn, will enable the feasibility of working as a midwife when fulfilled. Then midwives will have the possibility of developing their professional identity, grounded knowledge and professional courage to move within the different fields of midwives’ work, extending between normal to medicalised birth and being autonomous and regulated. This, in turn, may enable a professional building, characterised by a sustainable work situation with the possibility of being able to mobilise salutogenic resistance resources. This needs to be studied further in future research.

### Limitations and strengths

Purposive sampling was used, aiming for a variation in age, time as a midwife and work experience in the current workplace. This turned out to be more difficult than expected. One limitation of this study was that the majority of midwives interested in participating had long work experience. However, when comparing the coding of the different interviews, we could not see any systematic differences between the midwives with long vs. short experiences connected to the main concern. So, the goal of having a wide variety of work experience was shown to be overrated.

Another possible limitation was that the inclusion criteria might have created a sampling bias. The first criterion, being able to understand and speak Swedish, did not lead to sampling bias due to the fact that every midwife had to adhere to that criterion to be able to work on the labour ward. The last criterion, having at least 1 year of work experience as a midwife on the labour ward, was decided on because the first year is assumed an evolutionary period during which it is presumed that it is difficult to reflect on the work situation.

A constant comparative CGT was used to ensure the credibility of the results in this study [45]. Four of the authors are midwives with experience of labour ward work, which is a strength. However, this could lead to interpretive bias due to a pre-understanding of the interviews, context and emerged theory. This potential bias is counterbalanced by the fact that two of the authors are not midwives but operate in other fields, which enables another understanding of the material, context and analysis. The pre-understanding was continuously discussed during the whole process.

Another strength is that the results focus on salutogenic health-promoting facilitative conditions in midwives’ work situations and therefore on what retains them in their work on labour wards – an area that has scarcely been researched.

CGT should be abstracted from time, place and people [25] and is considered to be valid in another similar context, even if the study is done within a certain geographic area. The results in this study could therefore be valid in similar labour ward contexts where midwives work autonomously and is responsible for the professional domain of normal births. Although the strong connection to the profession probably exists regardless of organisation, country or context.

To review the validity and reliability of this study, we used the CGT criteria: fit, relevance, workability and modifiability [25]. During the analysis, a constant comparison of the concepts and the empirical data was undertaken, which led to a modification of the concepts so that they fitted the events they represented. This study seems to have relevance in the area of salutogenic health-promoting facilitative conditions in midwives’ work situations on labour wards since it evoked recognition by clinical colleagues when presented to them prior to publication. The professional courage to create a pathway within midwives’ fields of work represents the CGT concept of “workability” and the way in which midwives solve their main concern: *Feasibility of working as a midwife*. However, the emergent prerequisite conditions needed to be fulfilled to be able to attain that level of reflexivity. The theory of professional courage to create a pathway within midwives’ fields of work was modified during the analytical process. The generated substantive theory is also modifiable if new data were to be collected from other labour wards or other fields of practice in future studies [45]. There is a need for further research about primarily salutogenic but also pathogenic aspects of midwives’ work situations to ensure health-promoting working lives for midwives.

## Conclusion and clinical implications

Midwives requires a professional courage to create their own pathway within midwives’ fields of work to be able to navigate and move freely depending on what actions are needed in a particular work situation. Professional courage could be seen as a resistance resource, enabling midwives to become resilient when dealing with the unpredictable work situation. However, there are vital organisational preconditions that need to be fulfilled for workplaces to facilitate health-promotion and a sustainable work situation. Consequently, when an organisation provide organisational resources, visualise midwifery and provide a reflective and learning environment, it was feasible to be working as a midwife in a health-promoting way, which was the midwives’ main concern. When these preconditions were met, it led to positive consequences, such as professional identity and grounded knowledge that made it possible to achieve a professional courage. This was, in turn, the foundation for a sustainable working life.

The theory of attained *Professional courage to create a pathway within midwives’ fields of work* can be used to foster a health-promoting and sustainable work situation for midwives that is possible if the preconditions are met by the organisation. It could also as be used as a foundation for dialogue about the work situation and the philosophical differences in the way midwifery is viewed in the clinical setting. The described health-promoting facilitative conditions in the four-stage process should be strengthened at both the organisational, group and personal levels, and be emphasised even during the education of professionals on the labour ward. The theory could also be used in interventions to strengthen the work environment on labour wards and to enable the organisation to make use of the midwifery professions’ skills and knowledge. A health-promoting work situation could be a key factor to enable the retention of midwives in birthing care and not impoverish the midwifery profession and knowledge that is required for sustainable women-centred care. Improved occupational health, job satisfaction and well-being of midwives ought to be cost-effective for the organisation and society at large.

## Data Availability

The dataset generated and analysed during the current study is not publicly available as individual privacy could be compromised, but it is available from the corresponding author on reasonable request.

## Abbreviations

SOC: Sense of Coherence
CGT: Classical Grounded Theory
GDPR: General Data Protection Regulation
